# Constipation prevalence and its association with kidney function: a large nationwide Japanese health check-up cohort study

**DOI:** 10.1093/ckj/sfag082

**Published:** 2026-03-11

**Authors:** Keita Hirano, Keiichi Sumida, Shingo Fukuma

**Affiliations:** Human Health Sciences, Kyoto University Graduate School of Medicine, Kyoto, Japan; Department of Epidemiology, Disease Control and Prevention, Hiroshima University Graduate School of Biomedical and Health Sciences, Hiroshima, Japan; Department of Epidemiology, Disease Control and Prevention, Hiroshima University Graduate School of Biomedical and Health Sciences, Hiroshima, Japan; Division of Nephrology, Department of Medicine, University of Tennessee Health Science Center, Memphis, TN, USA; Nephrology Section, Veterans Affairs Greater Los Angeles Healthcare System, Los Angeles, CA, USA; David Geffen School of Medicine at the University of California Los Angeles, Los Angeles, CA, USA; Human Health Sciences, Kyoto University Graduate School of Medicine, Kyoto, Japan; Department of Epidemiology, Disease Control and Prevention, Hiroshima University Graduate School of Biomedical and Health Sciences, Hiroshima, Japan

**Keywords:** chronic kidney disease, constipation, epidemiology, laxatives, prescribing patterns

## Abstract

**Background:**

Constipation is one of the most common gastrointestinal disorders in the general population. However, its prevalence in patients with chronic kidney disease (CKD) and its association with kidney function have not been comprehensively examined.

**Methods:**

Using nationwide annual health check-ups and medical claims data collected in Japan from 2015–2023, we identified a total of 903 984 person-year observations from 217 734 unique individuals aged 20–74 years with available estimated glomerular filtration rate (eGFR) data. We evaluated the prevalence of constipation, defined by diagnostic codes or laxative use, and patterns of laxative prescriptions overall and across eGFR categories. Multivariable logistic regression models were used to assess the cross-sectional association between eGFR and constipation, adjusting for demographics, vital signs, comorbidities, and medication use.

**Results:**

Participants were 49.5 (SD, 10.4) years old, 72% were male, and 12.1% had diabetes. Among the 903 984 person-year observations, 87,861 (9.7%) had constipation. The prevalence of constipation was higher with lower eGFR, with 9.4%, 13.1%, 20.3%, 25.3%, and 45.2% in participants with eGFR ≥60, 45–59, 30–44, 15–29, and <15 ml/min/1.73 m², respectively. Laxative prescription patterns differed by CKD stages, with less use of magnesium salts and greater use of novel agents in more advanced CKD stages. After multivariable adjustment, compared with eGFR ≥60 ml/min/1.73 m², lower eGFR was significantly associated with a higher odds of constipation in a graded manner [adjusted odds ratios (95% CI), 1.07 (1.04–1.11), 1.14 (1.03–1.25), 1.47 (1.20–1.79), and 2.44 (1.84–3.22), respectively].

**Conclusion:**

Constipation was prevalent in patients with CKD. Lower kidney function was independently associated with higher odds of constipation, with the highest odds seen in the most advanced stage of CKD. These findings highlight the real-world burden of constipation across the CKD spectrum and suggest a potential pathophysiological link between constipation and impaired kidney function.

KEY LEARNING POINTS **What was known:**

- Constipation is one of the most common gastrointestinal disorders in the general population.
- The prevalence of constipation in patients with chronic kidney disease (CKD) and its association with kidney function have not been comprehensively examined.

**This study adds:**

- In a large nationwide cohort, the prevalence of constipation was higher in more advanced CKD stages, reaching nearly 50% of patients in stage 5.
- Laxative prescription patterns differed by CKD stage, with less magnesium salt use and greater use of newer agents in more advanced stages.
- Lower kidney function is significantly associated with higher odds of constipation, independent of demographics, comorbidities, and medication use.

**Potential impact:**

- Constipation should be recognized as a common complication across the full spectrum of kidney diseases.
- Our findings highlight the need for routine screening and individualized therapeutic strategies to optimize constipation management in the CKD population.
- Given the independent association between lower kidney function and constipation, interventions targeting either condition may help improve the other outcome.

## INTRODUCTION

Chronic kidney disease (CKD) is a growing public health concern because of its increasing prevalence and strong association with cardiovascular disease, end-stage kidney disease (ESKD), and mortality [1, 2]. Although several risk factors for CKD, including diabetes mellitus, hypertension, obesity, and smoking, among others, are well established, identifying additional comorbid conditions relevant to primary care may help reduce the burden of adverse outcomes and improve patient-centered management of CKD.

Constipation is a prototypical functional gastrointestinal disorder and one of the most prevalent conditions encountered in primary care [3]. Approximately 30% of the general population experiences constipation during their lifetime [4], with older adults and women being the most affected [5, 6]. Chronic constipation not only impairs health-related quality of life and work productivity but also imposes a substantial economic burden on healthcare systems [7, 8]. Recent observational studies have demonstrated that constipation is associated with a higher risk of adverse clinical outcomes, including incident cardiovascular disease, CKD, and ESKD [9–13], potentially mediated in part by systemic inflammation and altered gut microbiota [12].

Among patients with CKD, the symptom burden of constipation has been widely recognized. It has been attributed, at least in part, to shared risk factors for both conditions, including advanced age, dietary restrictions (e.g. limited fluid intake), comorbidities (e.g. diabetes mellitus), and the use of certain medications (e.g. phosphate binders) [14]. CKD-specific factors, including the accumulation of uremic toxins and electrolyte imbalances that impair gastrointestinal motility, may also contribute. Despite the clinical relevance of constipation in CKD, most studies assessing its prevalence have focused on patients with ESKD undergoing dialysis [15–19], and data on constipation among patients with non-dialysis-dependent CKD are limited [20]. A few recent studies have examined the prevalence of constipation across CKD stages and reported a higher prevalence in more advanced stages [21, 22]. However, these studies were largely descriptive and did not adequately account for the potential confounding effects of shared risk factors (e.g. older age) on the prevalence estimates across CKD stages. Therefore, it remains unclear whether lower kidney function is independently associated with a higher prevalence of constipation in patients with CKD.

In the present study, we sought to address this knowledge gap by leveraging a large, nationwide Japanese health check-up cohort with linked claims data. We hypothesized that constipation is prevalent among individuals with CKD and that impaired kidney function is independently associated with a greater likelihood of constipation in these patients. Our objectives were to comprehensively evaluate the prevalence of constipation across CKD stages and investigate the association between kidney function and constipation.

## MATERIALS AND METHODS

### Cohort definition and study design

We conducted a nationwide retrospective cohort study using an extensive longitudinal claims database in Japan that links annual health check-up records with medical claims at the individual level using an encrypted identifier. The database includes beneficiaries covered between April 2015 and March 2023.

**Figure 1:**
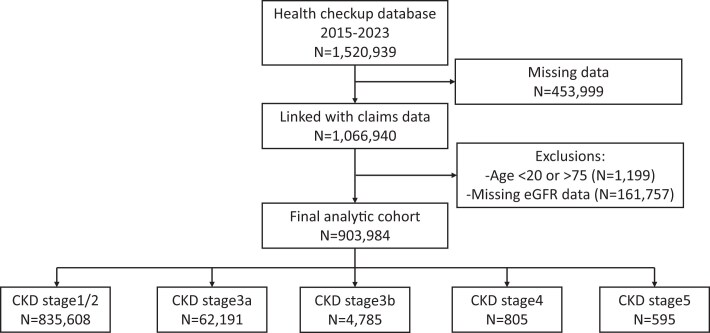
Flow diagram of cohort creation.

To construct the cohort, we analyzed repeated person-year observations and used clustered standard errors at the participant level. When multiple health check-ups were performed within a year, the earliest examination was retained for analysis. We included participants who underwent at least one health check-up and had claims data that could be successfully linked. We excluded person-years with insufficient information to link claims or missing essential eligibility fields, participants younger than 20 years or older than 75 years at the baseline check-up, and those without available eGFR data (Fig. 1). All records were anonymized and formally authorized by the insurance providers. The Institutional Review Board of Kyoto University approved all study procedures (approval no. R2913) and waived the requirement for informed consent owing to the use of deidentified data. This study was conducted according to the STROBE guidelines.

### Exposure and covariates

The exposure of interest was kidney function, defined as eGFR calculated using the Japanese-modified MDRD equation: eGFR = 194 × Cr^−1.094^ × Age^−0.287^ × (0.739 if female). Participants were categorized into five clinically aligned eGFR groups: ≥60, 45–59, 30–44, 15–29, and <15 ml/min/1.73 m^2^. Urine dipstick protein was categorized as negative, trace, and ≥1+. Covariates were collected at each annual health check-up. In cases where multiple health check-ups were recorded within the same fiscal year, data from the earliest examination that year were used to define the covariates. Demographic variables included age and sex, which were obtained from the insurer enrollment files. Body mass index (BMI) and systolic blood pressure (SBP) were measured during health check-ups. Comorbid conditions, including diabetes mellitus, hypertension, coronary heart disease, congestive heart failure, cerebrovascular disease, peripheral artery disease, dementia, chronic lung disease, rheumatologic disease, peptic ulcer disease, liver disease, malignancy, and HIV/AIDS, were identified from linked insurance claims using International Classification of Diseases (ICD)-10 codes, based on whether a related diagnosis was recorded at least once during the same fiscal year as the health check-up (Supplementary Table S1). Medication use, including oral iron supplements, potassium-lowering agents, phosphate binders, opioids, and antidepressants, was ascertained from prescription claims according to Anatomical Therapeutic Chemical codes if the prescription appeared at least once within the corresponding fiscal year (Supplementary Table S2). Socioeconomic indicators, such as household income and marital status, were obtained from administrative insurance data.

### Outcomes

The primary outcome of interest was constipation, which was ascertained from linked claims data and defined as the presence of a diagnostic code (Supplementary Table S1) or at least one prescription claim for any laxatives listed in Supplementary Table S2. The prevalence of constipation was calculated as the proportion of participants with constipation among those who underwent a health check-up in the same fiscal year and examined overall and across eGFR categories. The secondary outcome of interest was the proportion of individual laxative classes [i.e. magnesium salts, stimulant agents, bulk-forming agents, peripherally acting μ-opioid receptor antagonists (PAMORAs), novel agents (i.e. lubiprostone, linaclotide, and elobixibat), and non-magnesium-based osmotic agents] calculated as the ratio of the number of participants prescribed a laxative of interest to the number of participants prescribed any laxatives within the same fiscal year.

### Statistical analysis

We analyzed health check-up data collected between the fiscal years 2015 and 2023, with each fiscal year spanning from April to the following March. For each participant, all available records were organized by fiscal year, and when multiple check-ups were conducted within the same fiscal year, only the first record was used for analysis. Consequently, each participant contributed one health check-up record per fiscal year until the earliest of either the end of the follow-up or 31 March 2023. Because some participants contributed data across multiple years, we conducted a repeated cross-sectional analysis based on person-year observations to maximize the use of available annual records, with clustered standard errors used to account for within person correlation.

Baseline characteristics were summarized to describe the demographic, socioeconomic, comorbidity, laboratory, and medication profiles overall and across the kidney function categories. Continuous variables were reported as means with standard deviations, and categorical variables were reported as counts and percentages. The prevalence of constipation and the proportion of prescribed laxative classes were reported overall and by CKD stage, and their differences across stages were examined using chi-square test. To examine the association between kidney function and constipation, we fitted multivariable logistic regression models to estimate odds ratios (ORs) and 95% confidence intervals (CIs), with eGFR ≥60 ml/min/1.73 m^2^ as the reference group. The variables included in the multivariable adjustment were prespecified *a priori* based on clinical relevance and data availability. Model 1 included age, sex, and BMI. Model 2 was further adjusted for SBP and comorbid conditions, including diabetes mellitus, hypertension, coronary heart disease, congestive heart failure, cerebrovascular disease, peripheral artery disease, dementia, chronic lung disease, rheumatologic disease, peptic ulcer disease, liver disease, malignancy, and HIV/AIDS. Model 3 additionally included oral iron supplements, potassium-lowering agents, phosphate binders, opioids, and antidepressants.

We performed several sensitivity analyses to evaluate the robustness of our findings. To account for potential confounding by socioeconomic and temporal factors, we additionally included socioeconomic indicators (i.e. household income and marital status) and the calendar year of the health check-up in the multivariable adjustment. To address potential overcounting due to repeated person-year observations, we estimated the constipation prevalence by restricting the analysis to the first observation per individual within each eGFR category. Subgroup analyses were conducted according to sex, age (<65 vs ≥65 years), and BMI (<25 vs ≥25 kg/m^2^). Effect modification was evaluated by adding interaction terms and testing them with likelihood ratio tests, comparing models with and without the interaction term. All analyses were conducted using R (version 4.3.2; R Foundation for Statistical Computing, Vienna, Austria). A two-sided *P* < .05 was considered to be statistically significant.

## RESULTS

### Participant characteristics

Among the 903 984 person-years included in the final analytical cohort (Fig. 1), 835 608 (92.4%) had eGFR ≥60 ml/min/1.73 m^2^. The remaining participants had eGFR 45–59 (62 191 person-year observations, 6.9%); 30–44 (4 785 cases, 0.5%); 15–29 (805 cases, 0.1%) and <15 ml/min/1.73 m^2^ (595 cases, 0.1%) (Table 1). The mean age of the cohort was 49.5 ± 10.4 years; 26% were women, and 12.1% had diabetes mellitus. Compared to participants with preserved kidney function (i.e. eGFR ≥60 ml/min/1.73 m^2^), those with lower kidney function were more likely to be older and have comorbidities such as diabetes, hypertension, coronary heart disease, congestive heart failure, cerebrovascular disease, and peripheral artery disease. They also had greater proteinuria and lower high-density lipoprotein cholesterol levels and were more likely to be prescribed iron supplements, phosphate binders, opioids, antidepressants, and laxatives.

**Table 1:** Participants characteristics.

	eGFR category (ml/min/1.73 m^2^)					
	Overall	≥60	45–59	30–44	15–29	<15
	*n* = 903 984	*n* = 835 608	*n* = 62 191	*n* = 4 785	*n* = 805	*n* = 595
Age (SD), yr	49.5 (10.4)	48.8 (10.3)	57.7 (7.8)	60.6 (7.7)	57.5 (9.0)	56.3 (7.4)
Female, *n* (%)	235 421 (26.0)	234,757 (28.1)	518 (0.8)	79 (1.7)	36 (4.5)	31 (5.2)
BMI (SD), kg/m^2^	23.9 (3.9)	23.8 (3.9)	25.3 (3.5)	25.9 (4.1)	25.6 (4.5)	25.8 (4.7)
Systolic BP (SD), mmHg	123.2 (16.3)	122.8 (16.2)	127.7 (16.4)	129.2 (17.8)	131.4 (20.3)	137.4 (22.2)
Diastolic BP (SD), mmHg	76.6 (11.9)	76.3 (11.9)	80.5 (11.5)	79.9 (12.0)	80.1 (13.1)	80.1 (13.1)
Socioeconomic status						
Mean household income (SD), $	50 604 (21 405)	50 510 (21 198)	52 420 (23 784)	45 302 (22 899)	45 534 (22 935)	43 167 (18 895)
Married, *n* (%)	537 075 (59.4)	488 746 (58.5)	44 288 (71.2)	3 217 (67.2)	512 (63.6)	312 (52.4)
Comorbidities						
Diabetes mellitus, *n* (%)	109 106 (12.1)	91 854 (11.0)	14 210 (22.8)	2 214 (46.3)	467 (58.0)	361 (60.7)
Hypertension, *n* (%)	184 226 (20.4)	152 041 (18.2)	27 157 (43.7)	3 701 (77.3)	745 (92.5)	582 (97.8)
CHD, *n* (%)	5 409 (0.6)	4 156 (0.5)	1 031 (1.7)	142 (3.0)	47 (5.8)	33 (5.5)
CHF, *n* (%)	28 031 (3.1)	22 091 (2.6)	4 661 (7.5)	815 (17.0)	195 (24.2)	269 (45.2)
CVD, *n* (%)	33 907 (3.8)	28 209 (3.4)	4 804 (7.7)	673 (14.1)	115 (14.3)	106 (17.8)
PAD, *n* (%)	27 977 (3.1)	23 537 (2.8)	3 625 (5.8)	540 (11.3)	111 (13.8)	164 (27.6)
Dementia, *n* (%)	376 (0.0)	311 (0.0)	53 (0.1)	10 (0.2)	2 (0.2)	0 (0.0)
Chronic lung disease, *n* (%)	112 871 (12.5)	103 472 (12.4)	8 425 (13.5)	716 (15.0)	151 (18.8)	107 (18.0)
Rheumatologic disease, *n* (%)	9 693 (1.1)	8 711 (1.0)	819 (1.3)	118 (2.5)	30 (3.7)	15 (2.5)
Peptic ulcer disease, *n* (%)	55 140 (6.1)	48 486 (5.8)	5 596 (9.0)	718 (15.0)	175 (21.7)	165 (27.7)
Liver disease, *n* (%)	94 919 (10.5)	83 725 (10.0)	9 773 (15.7)	1 132 (23.7)	180 (22.4)	109 (18.3)
Malignancies, *n* (%)	37 206 (4.1)	31 932 (3.8)	4 376 (7.0)	703 (14.7)	118 (14.7)	77 (12.9)
HIV/AIDS, *n* (%)	297 (0.0)	237 (0.0)	52 (0.1)	5 (0.1)	3 (0.4)	0 (0.0)
Laboratory data						
eGFR (SD), ml/min/1.73 m^2^	84.3 (20.9)	86.9 (19.5)	55.2 (3.7)	40.3 (3.8)	23.7 (4.3)	7.6 (3.4)
Proteinuria, *n* (%)						
Negative, *n* (%)	793 824 (87.8)	739 477 (88.5)	51 311 (82.5)	2 861 (59.8)	149 (18.5)	26 (4.4)
Trace, *n* (%)	79 032 (8.7)	71 983 (8.6)	6 409 (10.3)	559 (11.7)	60 (7.5)	21 (3.5)
Positive, *n* (%)	31 128 (3.4)	24 148 (2.9)	4 471 (7.2)	1 365 (28.5)	596 (74.0)	548 (92.1)
HbA1c, %	5.6 (0.7)	5.6 (0.7)	5.8 (0.7)	6.1 (1.0)	6.1 (1.1)	5.8 (1.0)
FPG (SD), mg/dl	96.8 (19.2)	96.3 (18.9)	102.4 (19.8)	109.3 (28.7)	110.8 (31.7)	105.5 (30.6)
HDL cholesterol (SD), mg/dl	62.0 (16.8)	62.5 (16.9)	57.2 (14.9)	54.1 (14.6)	53.6 (16.4)	52.7 (17.5)
Triglycerides (SD), mg/dl	116.3 (94.5)	114.7 (94.5)	134.0 (91.1)	153.7 (104.7)	170.8 (131.6)	133.3 (104.4)
Medication use						
Iron supplement, *n* (%)	3 740 (0.4)	3 442 (0.4)	63 (0.1)	17 (0.4)	9 (1.1)	209 (35.1)
Phosphate binder, *n* (%)	240 (0.0)	120 (0.0)	14 (0.0)	12 (0.3)	16 (2.0)	78 (13.1)
Opioid, *n* (%)	36 089 (4.0)	32 918 (3.9)	2 838 (4.6)	249 (5.2)	56 (7.0)	28 (4.7)
Antidepressant, *n* (%)	2 740 (0.3)	2 484 (0.3)	219 (0.4)	28 (0.6)	5 (0.6)	4 (0.7)
Laxative, *n* (%)	49 280 (5.5)	43 841 (5.2)	4 806 (7.7)	472 (9.9)	89 (11.1)	72 (12.1)

Note: values are presented as means (standard deviations) or numbers (percentages). Proteinuria was defined as negative, trace, and 1+ or greater (positive dipstick protein (≥1+) or equivalent laboratory measurement). Socioeconomic status was assessed using the mean household income.

Abbreviations: BP, blood pressure; FPG, fasting plasma glucose; HbA1c, hemoglobin A1c; HDL, high-density lipoprotein; CHD, coronary heart disease; CHF, congestive heart failure; CVD, cerebrovascular disease; PAD, peripheral artery disease.

### Constipation prevalence and laxative prescription patterns across CKD stages

**Figure 2:**
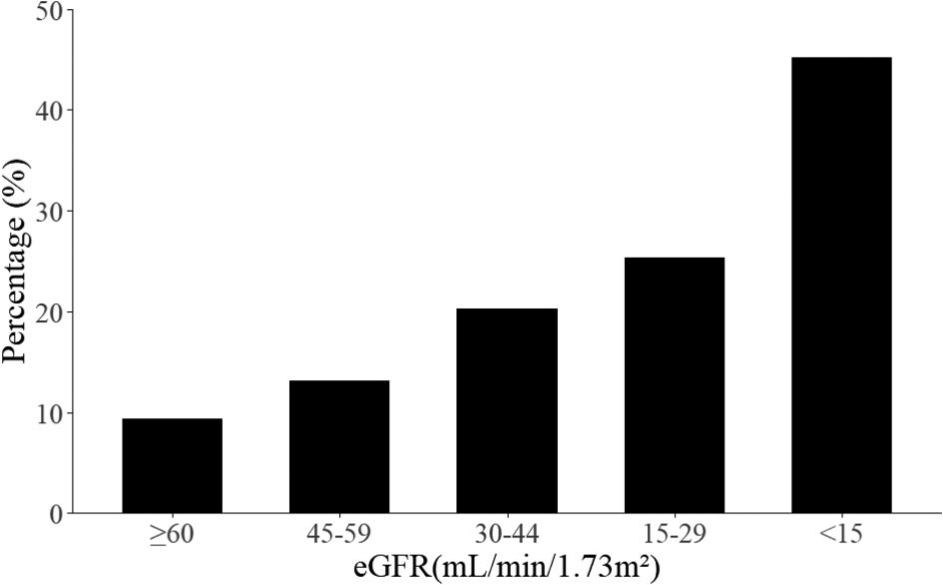
Prevalence of constipation in CKD stages. Proportion of participants with constipation was higher with lower eGFR levels in a graded manner.

Overall, constipation was present in 87 861 of the 903 984 person-years observations (9.7%). As shown in Fig. 2, the prevalence of constipation was higher with lower eGFR levels in a graded manner, with values of 9.4%, 13.1%, 20.3%, 25.3%, and 45.2% for eGFR ≥60, 45–59, 30–44, 15–29, and <15 mL/min/1.73 m², respectively. Constipation prevalence did not materially differ when further stratified by urine dipstick protein categories (negative, trace, and ≥1+) (Supplementary Fig. S1). In a sensitivity analysis restricted to the first observation per individual within each eGFR category, the prevalence of constipation was essentially unchanged from that of the primary analysis (Supplementary Fig. S2).

**Figure 3:**
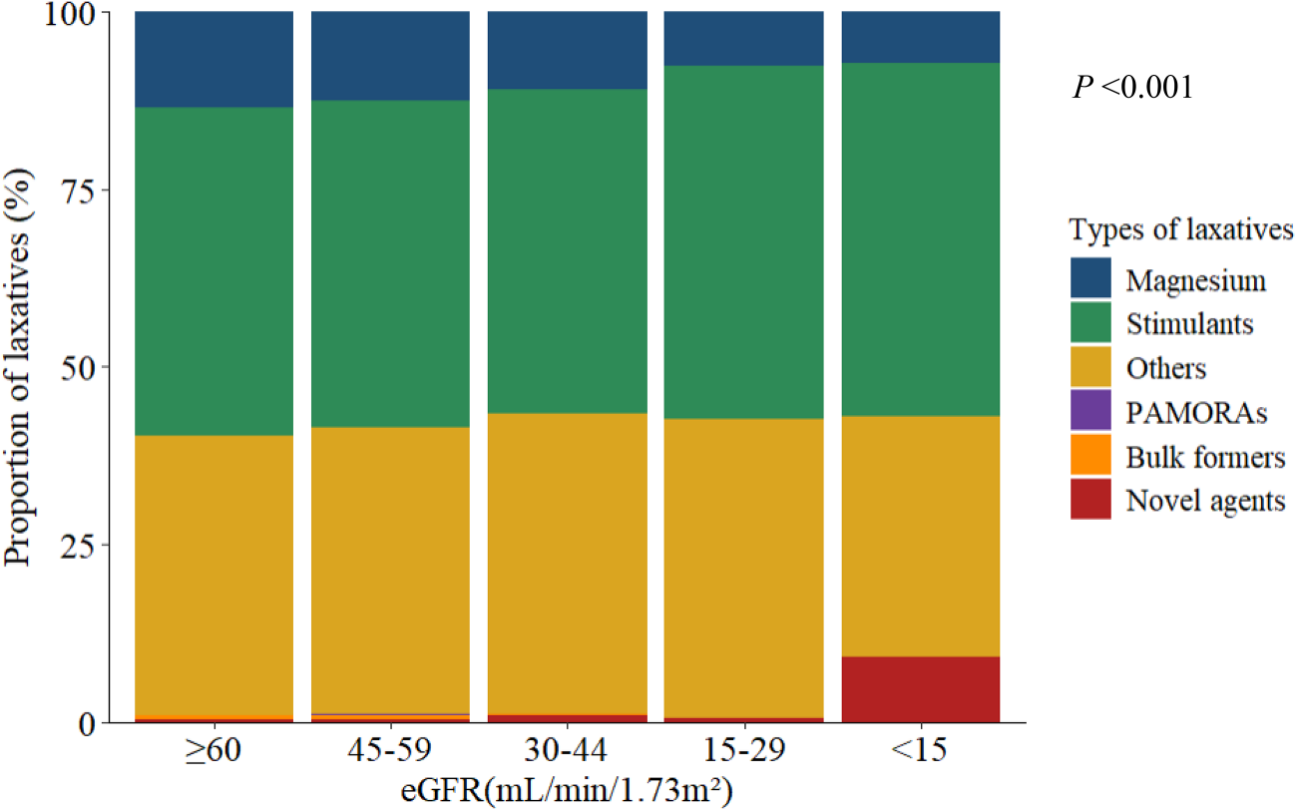
Laxative prescription patterns across CKD stages. The proportions of prescriptions for each laxative type are shown. The categories included magnesium salts, stimulant laxatives, bulk formers, non-magnesium-based osmotic agents (others), peripherally acting μ-opioid receptor antagonists (PAMORAs), and novel agents.

Among the 49 280 participants who were prescribed any class of laxatives, stimulants were the most commonly prescribed (45.9%), followed by non-magnesium-based osmotic agents (40.2%), magnesium salts (13.3%), bulk-forming agents (0.6%), novel agents (0.6%), and PAMORAs (0.1%). When the proportions of individual laxative classes were examined across CKD stages, distinct patterns of laxative prescriptions emerged, with statistically significant differences across stages (*P* < .001) (Fig. 3). While stimulant and non-magnesium-based osmotic agents were most commonly prescribed in all CKD stages, the prescription of magnesium salts was less common in more advanced CKD stages, with proportions of 13.6%, 12.5%, 11.0%, 7.7%, and 7.3% for eGFR ≥60, 45–59, 30–44, 15–29, and <15 ml/min/1.73 m², respectively. By contrast, the prescription of novel agents was more frequent in more advanced CKD, with the highest proportion (9.2%) observed in those with eGFR <15 ml/min/1.73 m². Bulk-forming agents and PAMORAs were infrequently prescribed at all stages (∼0.6% and 0.1%, respectively).

### Association between eGFR and constipation

Table 2 shows the association between eGFR and constipation in the study population. In the minimally adjusted model accounting for age, sex, and BMI, lower eGFR was significantly associated with higher odds of constipation in a graded manner [adjusted ORs (95% CI), 1.21 (1.18–1.25), 1.79 (1.67–1.93), 2.71 (2.30–3.19), and 7.20 (6.10–8.51) for eGFR 45–59, 30–44, 15–29, and <15 (vs. ≥60) ml/min/1.73 m², respectively; Model 1]. Although these associations were attenuated after further multivariable adjustment, the odds of constipation remained significantly and incrementally higher at lower eGFR levels, with the highest odds seen in the lowest eGFR category [adjusted ORs (95% CI), 1.07 (1.04–1.11), 1.14 (1.03–1.25), 1.47 (1.20–1.79), and 2.44 (1.84–3.22) for eGFR 45–59, 30–44, 15–29, and <15 (vs. ≥60) ml/min/1.73 m², respectively; model 3].

**Table 2:** ORs and 95% CIs of constipation associated with eGFR categories.

	eGFR category (ml/min/1.73 m^2^)				
	≥60	45–59	30–44	15–29	<15
Model 1^a^	Reference	1.21	1.79	2.71	7.20
		(1.18–1.25)	(1.67–1.93)	(2.30–3.19)	(6.10–8.51)
Model 2^b^	Reference	1.07	1.12	1.55	3.44
		(1.03–1.10)	(1.02–1.22)	(1.29–1.87)	(2.65–4.48)
Model 3^c^	Reference	1.07	1.14	1.47	2.44
		(1.04–1.11)	(1.03–1.25)	(1.20–1.79)	(1.84–3.22)

Note: values are presented as adjusted OR with 95% CI.

a Model 1 was adjusted for age, sex, and BMI.

b Model 2 was additionally adjusted for SBP and comorbidities (coronary heart disease, heart failure, cerebrovascular disease, peripheral artery disease, diabetes mellitus, hypertension, dementia, chronic lung disease, rheumatologic disease, peptic ulcer disease, liver disease, malignancies, and HIV/AIDS) in addition to the covariates of Model 1.

c Model 3 was further adjusted for medication use (oral iron supplements, potassium-lowering agents, phosphate binders, opioids, and antidepressants) in addition to the covariates in Model 2.

**Figure 4:**
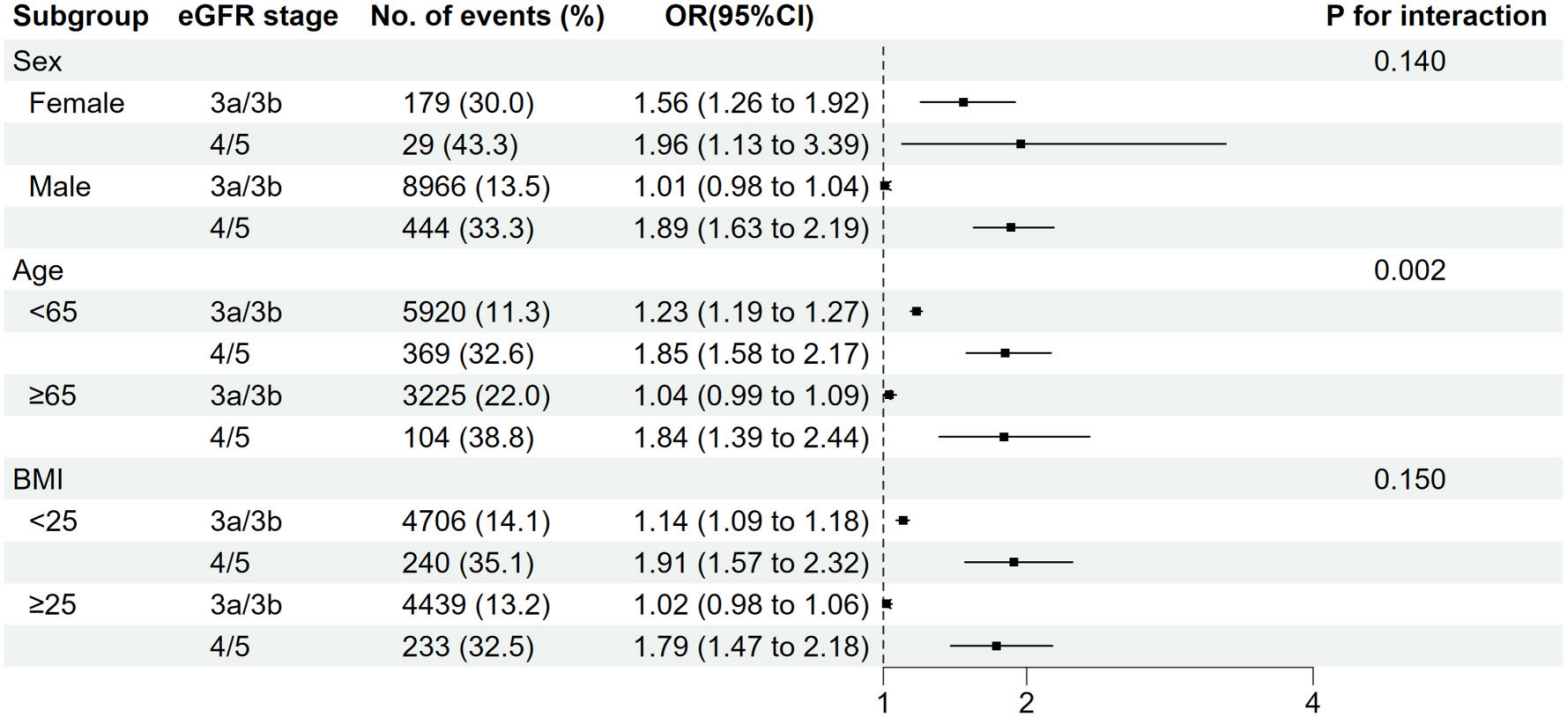
Association between eGFR and constipation in selected subgroups. All analyses were adjusted using Model 3, which included age, sex, BMI, SBP, comorbidities (diabetes mellitus, hypertension, coronary heart disease, heart failure, cerebrovascular disease, peripheral artery disease, dementia, chronic lung disease, rheumatologic disease, peptic ulcer disease, liver disease, malignancy, and HIV/AIDS), and medication use (oral iron supplements, potassium-lowering agents, phosphate binders, opioids, and antidepressants).

Similar results were observed in the sensitivity analyses. The associations between eGFR and constipation were similar after further adjustment for socioeconomic indicators or the calendar year of the health check-up (i.e. models S1 and S2 in Supplementary Table S3). In the subgroup analysis, a lower eGFR was consistently associated with a higher odds of constipation in the selected subgroups (Fig. 4). With a notable exception, the higher odds of constipation associated with moderate stages of CKD [i.e. eGFR 30–59 (vs. ≥60) ml/min/1.73 m²] were more evident among female participants, those aged <65 years, and those with BMI <25 than among their counterparts, with a statistically significant interaction observed only for age (*P* for interaction = .002).

## DISCUSSION

In this large nationwide cohort of Japanese adults with annual health check-ups and medical claims data, we found that the prevalence of constipation was higher in more advanced CKD stages and that laxative prescription patterns differed by CKD stage, with less use of magnesium salts and greater use of novel agents in more advanced stages. Furthermore, we found that lower kidney function was independently associated with higher odds of constipation, with the highest odds seen in the most advanced stage of CKD. Compared with participants with eGFR ≥60 ml/min/1.73 m², those with eGFR <15 ml/min/1.73 m² had ∼2.4-fold higher odds of constipation in the multivariable-adjusted models. The findings were robust to several sensitivity analyses.

Constipation is one of the most common gastrointestinal manifestations in patients with CKD [23]. However, most previous studies examining the prevalence of constipation in CKD have focused on the dialysis population, with a limited number of studies investigating the prevalence of constipation among individuals with non-dialysis-dependent CKD. Furthermore, these studies were often limited by small sample sizes and stage-specific prevalence estimates [14, 23], and few have comprehensively assessed the prevalence of constipation alongside laxative prescription patterns across the CKD spectrum. In a recent nationwide cohort of 39 293 Korean individuals with CKD diagnosed between 2012 and 2017, researchers examined the prevalence of constipation and laxative use across CKD stages. They reported a higher prevalence of constipation and greater laxative use in later stages of CKD [22]. Specifically, they showed that the prevalence of constipation was 15.9%, 16.5%, 17.4%, 29.9%, and 43.3% in CKD stages 1 through 5, respectively, accompanied by stage-specific variations in laxative prescribing patterns, characterized by less use of magnesium salts and bulking agents and greater use of lactulose in more advanced stages [22]. While our findings were generally consistent with these observations, the present study extends the existing evidence in three important ways. First, we examined a larger nationwide sample from the general Japanese population using more contemporary data collected between 2015 and 2023. Second, we provided a detailed, class-specific description of laxative use across CKD stages, including contemporary agents, thereby offering a more complete real-world picture of constipation management across the CKD spectrum; this also highlighted a reciprocal prescribing pattern in advanced CKD, characterized by greater use of novel laxative agents, such as peripherally acting PAMORAs and lubiprostone, and less frequent use of magnesium salts. Third, most importantly, we demonstrated an independent association between lower kidney function and constipation, even after comprehensive adjustment for demographic factors, vital signs, comorbidities, medication use, and socioeconomic status.

Although our observational study cannot establish a causal relationship, several plausible mechanisms may underlie the observed association between lower kidney function and constipation. First, an altered gut microbiota is increasingly recognized as an important shared factor in the pathophysiology of both constipation and CKD [12, 14, 24, 25]. Patients with CKD, particularly those with advanced stages, have been shown to exhibit significant alterations in the gut microbiota, which contribute to adverse clinical outcomes, such as CKD progression and cardiovascular disease [11, 26]. Altered gut microbiota and gastrointestinal dysmotility are closely interrelated and exert reciprocal effects on one another; as a consequence, constipation is often regarded as a clinical manifestation of altered gut microbiota [26]. In addition, metabolic and endocrine disturbances associated with CKD may directly impair colonic function [27]. The accumulation of uremic toxins and electrolyte imbalances, such as hypercalcemia and hypomagnesemia, can disrupt smooth muscle contractility and neural regulation of the gastrointestinal tract [28–31]. Furthermore, autonomic dysfunction, which is prevalent in advanced CKD, may further compromise gastrointestinal motility [32].

Our results have several important clinical implications. First, the stepwise increase in the prevalence of constipation with advancing CKD indicates that constipation should be recognized as a common complication across the full spectrum of kidney diseases, not only among patients receiving dialysis. This recognition highlights the need for routine screening and proactive assessment of bowel symptoms as an integral part of CKD care, particularly among patients with advanced CKD stages. Second, while the observed laxative prescribing patterns across CKD stages suggest that clinicians have already adjusted their treatments to balance efficacy with safety, given the current reliance on individual clinicians’ discretion and the close link between constipation and CKD, our findings underscore the need for evidence-based guidelines to optimize constipation management in the CKD population. Third, the independent association between kidney function and constipation suggests that constipation should be viewed as a clinical manifestation of CKD itself rather than merely a consequence of concomitant conditions related to CKD, such as aging, comorbidities, and frequent use of constipation-inducing medications. Therefore, early recognition and effective management of constipation in CKD may not only enhance patients’ quality of life and adherence to therapy but also potentially improve clinical outcomes, particularly in light of previous studies demonstrating an association between constipation and adverse clinical outcomes [13, 33].

This study had several limitations. First, constipation was defined using diagnostic codes and prescription records rather than standardized symptom questionnaires, such as the Rome IV criteria. As a consequence, some degree of misclassification is possible, as individuals with milder symptoms who did not seek medical attention and instead self-managed constipation with over-the-counter laxatives may have been underrecognized. This may also have introduced differential ascertainment by health status, since patients with advanced CKD and multiple comorbidities have more frequent healthcare contact and are therefore more likely to receive a recorded diagnosis or prescription, potentially inflating the observed association between lower kidney function and constipation. Furthermore, given that stimulant (e.g. sennosides and bisacodyl) and magnesium-based agents are the predominant over-the-counter laxatives in Japan [34] and are likely used to self-manage mild constipation among patients with earlier (vs. more advanced) CKD stages for the reason described above, our prescription-based data may underestimate both constipation prevalence and conventional laxative use in earlier CKD stages, potentially making the observed “reciprocal prescribing pattern” more pronounced in reality than reported. In addition, our claims-based definition cannot distinguish clinically heterogeneous constipation subtypes (e.g. functional constipation, IBS-C, or symptom-based phenotypes), which may differ in underlying mechanisms and clinical implications; thus, our prevalence estimates and associations should be interpreted as reflecting a composite, healthcare-recorded constipation outcome rather than specific constipation phenotypes. Nevertheless, our approach to defining constipation is consistent with prior large-scale epidemiological studies [13, 22] and enables standardized ascertainment within a real-world clinical database. Second, albuminuria data were not available in our dataset; therefore, misclassification of CKD staging is possible, particularly in early CKD stages with preserved eGFR [35–37]. However, further stratification of CKD stages by urine dipstick protein categories, a practical surrogate for albuminuria severity, did not reveal material differences in constipation prevalence. Third, although we adjusted for a wide range of potential confounders, including demographic, clinical, pharmacological, and socioeconomic covariates, the possibility of unmeasured or residual confounding (e.g. dietary fiber intake, hydration status, and patient-reported lifestyle behaviors) cannot be fully excluded. Moreover, some medications included in our adjustment models may lie on the causal pathway between reduced kidney function and constipation, which could result in partial overadjustment and attenuation of the estimated association. Nevertheless, despite this potential attenuation, the association between kidney function and constipation remained statistically significant in the fully adjusted model. Fourth, our study was cross-sectional, which could not establish the temporality of the association between kidney function and constipation or infer a causal relationship. Further studies, including clinical trials, are needed to clarify the temporality of constipation development as kidney function decline, to elucidate the causal pathways underlying these relationships, and to evaluate whether targeting either condition may improve the other outcome. In addition, analyses using person-year observations may have resulted in some inflation of prevalence estimates, as individuals with chronic constipation could contribute multiple records within the same eGFR category. However, prevalence estimates obtained by restricting the analysis to the first observation per individual were consistent with the primary results, indicating that our findings were not materially influenced by repeated observations. Finally, our cohort was derived from a Japanese health check-up population, which may limit the generalizability of our findings to populations with different healthcare systems, dietary patterns, or genetic backgrounds. Nevertheless, our large population-based cohort enabled robust CKD stage-specific estimates of the association between kidney function and constipation.

In conclusion, in this large nationwide population-based cohort, constipation was more prevalent in advanced stages of CKD, ranging from ∼10% in stages 1–2 to nearly 50% in stage 5. Lower kidney function was independently associated with a higher risk of constipation, suggesting that constipation may represent an intrinsic complication of CKD rather than merely a consequence of aging, comorbid conditions, or adverse drug effects. These findings underscore the need to recognize constipation as a clinically important burden in CKD care and to develop evidence-based strategies for its prevention and management in patients with CKD.

## Supplementary Material

sfag082_Supplemental_File

## Data Availability

Owing to the nature of this research, patient data were not shared publicly due to participants’ privacy; therefore, supporting data are not available.
