# Supplementary material for: Constipation prevalence and its association with kidney function: a large nationwide Japanese health check-up cohort study
Source: Clin Kidney J. 2026 Mar 11;19(5):sfag082. doi: 10.1093/ckj/sfag082 (PMC13147125; doi:10.1093/ckj/sfag082)
Supplement: sfag082_Supplemental_File [file sfag082_supplemental_file.docx]

**Supplementary Materials**

**Table of Contents**

**Supplemental Table 1.** Definitions of comorbidities based on International Classification of Diseases (ICD)-10 codes

**Supplemental Table 2.** Definitions of medications based on Anatomical Therapeutic Chemical (ATC) codes

**Supplemental Table 3.** Odds ratios and 95% confidence intervals of constipation associated with estimated glomerular filtration rate (eGFR) categories after further adjustment for socioeconomic status or calendar year of health check-up

**Supplemental Figure 1.** Constipation prevalence by eGFR category further stratified by urine dipstick protein category

**Supplemental Figure 2.** Constipation prevalence by eGFR category using the first observation per individual

**Supplementary Table 1.** Definitions of comorbidities based on International Classification of Diseases (ICD)-10 codes

| **Comorbidity** | **ICD-10 Codes** |
| --- | --- |
| Constipation | K59.0 |
| Coronary heart disease | I21.x, I22.x, I25.2 |
| Congestive heart failure | I09.9, I11.0, I13.0, I13.2, I25.5, I42.0, I42.5–I42.9, I43.x, I50.x, P29.0 |
| Peripheral artery disease | I70.x, I71.x, I73.1, I73.8, I73.9, I77.1, I79.0, I79.2, K55.1, K55.8, K55.9, Z95.8, Z95.9 |
| Cerebrovascular disease | G45.x, G46.x, H34.0, I60.x–I69.x |
| Dementia | F00.x–F03.x, F05.1, G30.x, G31.1 |
| Chronic lung disease | I27.8, I27.9, J40.x–J47.x, J60.x–J67.x, J68.4, J70.1, J70.3 |
| Rheumatic disease | M05.x, M06.x, M31.5, M32.x–M34.x, M35.1, M35.3, M36.0 |
| Peptic ulcer disease | K25.x–K28.x |
| Liver disease | B18.x, K70.0–K70.3, K70.9, K71.3–K71.5, K71.7, K73.x, K74.x, K76.0, K76.2–K76.4, K76.8, K76.9, Z94.4 |
| Diabetes mellitus | E10.x-E14.x |
| Renal disease | I12.0, I13.1, N03.2–N03.7, N05.2–N05.7, N18.x, N19.x, N25.0, Z49.0–Z49.2, Z94.0, Z99.2 |
| Malignancies | C00.x–C26.x, C30.x–C34.x, C37.x–C41.x, C43.x, C45.x–C58.x, C60.x–C76.x, C81.x–C85.x, C88.x, C90.x–C97.x |
| AIDS/HIV | B20.x–B22.x, B24.x |
|  |  |

**Supplementary Table 2.** Definitions of medications based on Anatomical Therapeutic Chemical (ATC) codes

| **Medications** | **ATC code** |
| --- | --- |
| **Laxatives** |  |
| Magnesium salts | A06AD04, A06AD19 |
| Peripherally acting μ-opioid receptor  　antagonists (PAMORAs) | A06AH05 |
| Stimulant agents | A06AB02, A06AB05, A06AB06, A06AB56, A06AB07, A06AB57, A06AB08, A06AB58, A06AG04, A06AG06, A06AX02 |
| Bulk forming agents | A06AC06, A06AC08 |
| Novel agents | A06AX03, A06AX04, A06AX09 |
| Non-magnesium-based osmotic agents | A06AD10, A06AD17, A06AD11, A06AD18, A06AD12, A06AD65, B05CX02, V04CC01 |
| **Iron supplements** | B03AA01, B03AA02, B03AA03, B03AA04, B03AA05, B03AA06, B03AA07, B03AA08, B03AA09, B03AA10, B03AA11, B03AA12, B03AB01, B03AB02, B03AB03, B03AB04, B03AB05, B03AB07, B03AB08, B03AB09, B03AB10, B03AC, B03AD01, B03AD02, B03AD03, B03AD04, B03AD05, B03AE01, B03AE02, B03AE03, B03AE04, B03AE10 |
| **Phosphate binders** | V03AE01, V03AE02, V03AE03, V03AE04, V03AE05, V03AE06, V03AE07, V03AE08, V03AE09, V03AE10 |
| **Opioids** | N02AA01, N02AA02, N02AA03, N02AA04, N02AA05, N02AA08, N02AA10, N02AA11, N02AA51, N02AA53, N02AA55, N02AA56, N02AA58, N02AA59, N02AA79, N02AB01, N02AB02, N02AB03, N02AB52, N02AB72, N02AC01, N02AC03, N02AC04, N02AC05, N02AC52, N02AC54, N02AC74, N02AD01, N02AD02, N02AD51, N02AE01, N02AF01, N02AF02, N02AG01, N02AG02, N02AG03, N02AG04, N02AJ01, N02AJ02, N02AJ03, N02AJ06, N02AJ07, N02AJ08, N02AJ09, N02AJ13, N02AJ14, N02AJ15, N02AJ16, N02AJ17, N02AJ18, N02AJ19, N02AJ22, N02AJ23, N02AX01, N02AX02, N02AX03, N02AX05, N02AX06, N02AX07, N02AX51 |
| **Antidepressants** | N06AA01, N06AA02, N06AA03, N06AA04, N06AA05, N06AA06, N06AA07, N06AA08, N06AA09, N06AA10, N06AA11, N06AA12, N06AA13, N06AA14, N06AA15, N06AA16, N06AA17, N06AA18, N06AA19, N06AA21, N06AA23, N06AB02, N06AB03, N06AB04, N06AB05, N06AB06, N06AB07, N06AB08, N06AB09, N06AB10, N06AF01, N06AF02, N06AF03, N06AF04, N06AF05, N06AF06, N06AG02, N06AG03, N06AX01, N06AX02, N06AX03, N06AX04, N06AX05, N06AX06, N06AX07, N06AX08, N06AX09, N06AX10, N06AX11, N06AX12, N06AX13, N06AX14, N06AX15, N06AX16, N06AX17, N06AX18, N06AX19, N06AX20, N06AX21, N06AX22, N06AX23, N06AX24, N06AX25, N06AX26, N06AX27, N06AX28, N06AX29, N06AX31, N06AX62 |

**Supplementary Table 3.** Odds ratios and 95% confidence intervals of constipation associated with estimated glomerular filtration rate (eGFR) categories after further adjustment for socioeconomic status or calendar year of health check-up

|  | **eGFR category (mL/min/1.73 m^2^)** | | | | |
| --- | --- | --- | --- | --- | --- |
|  | **≥60** | **45-59** | **30-44** | **15-29** | **<15** |
| Model S1^a^ | Reference | 1.07  (1.04-1.10) | 1.13  (1.03-1.24) | 1.46  (1.20-1.78) | 2.44  (1.84-3.22) |
| Model S2^b^ | Reference | 1.06  (1.03-1.09) | 1.12  (1.02-1.23) | 1.46  (1.20-1.79) | 2.41  (1.82-3.20) |

^a^Model S1 was further adjusted for socioeconomic status (per capita income and marital status) in addition to the covariates of Model 3.

^b^Model S2 was further adjusted for the calendar year of the health check-up, in addition to the covariates in Model 3.

Values are presented as adjusted odds ratios (95% confidence intervals).

**Supplemental Figure 1.** Constipation prevalence by eGFR category further stratified by urine dipstick protein category


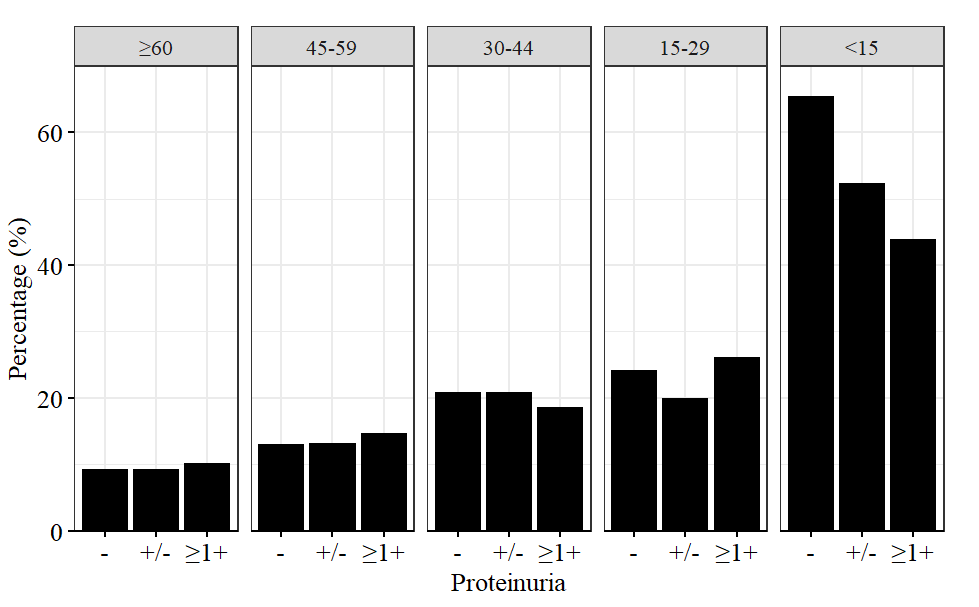


|  | eGFR(mL/min/1.73m²) | | | | |
| --- | --- | --- | --- | --- | --- |
| urine dipstick protein | ≥60 | 45-59 | 30-44 | 15-29 | <15 |
| Negative | 69074 (9.3) | 6667 (13.0) | 598 (20.9) | 36 (24.2) | 17 (65.4) |
| Trace | 6707 (9.3) | 847 (13.2) | 117 (20.9) | 12 (20.0) | 11 (52.4) |
| Positive | 2463 (10.2) | 661 (14.8) | 255 (18.7) | 156 (26.2) | 241 (44.0) |

Urine dipstick protein was categorized as negative, trace, and 1+ or greater (positive). In tables and figures, these categories are shown as -, +/-, and ≥1+, respectively.

**Supplemental Figure 2.** Constipation prevalence by eGFR category using the first observation per individual


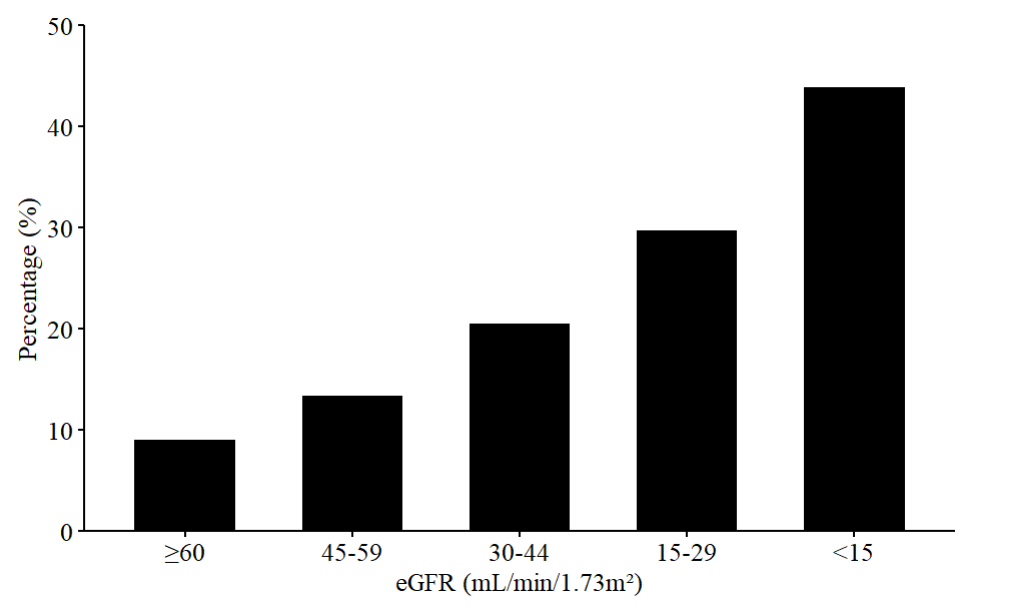


| No. of events(%) of constipation | | | | |
| --- | --- | --- | --- | --- |
| eGFR(mL/min/1.73m²) | | | | |
| ≥60 | 45-59 | 30-44 | 15-29 | <15 |
| 18,561(9.0) | 1,372(13.3) | 171(20.5) | 47(29.7) | 57(43.8) |
